# Evaluating alignment quality between iconic language and reference terminologies using similarity metrics

**DOI:** 10.1186/1472-6947-14-17

**Published:** 2014-03-11

**Authors:** Nicolas Griffon, Gaetan Kerdelhué, Lina F Soualmia, Tayeb Merabti, Julien Grosjean, Jean-Baptiste Lamy, Alain Venot, Catherine Duclos, Stefan J Darmoni

**Affiliations:** 1 CISMeF, Rouen University Hospital, Normandy & TIBS, LITIS EA 4108, Institute for Research and Innovation in Biomedicine, Rouen, France; 2 INSERM, U1142, LIMICS, F-75006 Paris, France; 3 Sorbonne Universités, UPMC Univ Paris 06, UMR_S 1142, LIMICS, F-75006 Paris, France; 4 Université Paris 13, Sorbonne Paris Cité, LIMICS, (UMR_S 1142), F-93430 Villetaneuse, France

**Keywords:** Terminology as topic, International classification of diseases, Medical subject headings, Vocabulary, Controlled, Alignment, Iconic language, Compositional language, Semantic distances, Inter-alignment agreement

## Abstract

**Background:**

Visualization of Concepts in Medicine (VCM) is a compositional iconic language that aims to ease information retrieval in Electronic Health Records (EHR), clinical guidelines or other medical documents. Using VCM language in medical applications requires alignment with medical reference terminologies. Alignment from Medical Subject Headings (MeSH) thesaurus and International Classification of Diseases – tenth revision (ICD10) to VCM are presented here. This study aim was to evaluate alignment quality between VCM and other terminologies using different measures of inter-alignment agreement before integration in EHR.

**Methods:**

For medical literature retrieval purposes and EHR browsing, the MeSH thesaurus and the ICD10, both organized hierarchically, were aligned to VCM language. Some MeSH to VCM alignments were performed automatically but others were performed manually and validated. ICD10 to VCM alignment was entirely manually performed. Inter-alignment agreement was assessed on ICD10 codes and MeSH descriptors, sharing the same Concept Unique Identifiers in the Unified Medical Language System (UMLS). Three metrics were used to compare two VCM icons: binary comparison, crude Dice Similarity Coefficient (DSC_*crude*_), and semantic Dice Similarity Coefficient (DSC_*semantic*_), based on Lin similarity. An analysis of discrepancies was performed.

**Results:**

MeSH to VCM alignment resulted in 10,783 relations: 1,830 of which were manually performed and 8,953 were automatically inherited. ICD10 to VCM alignment led to 19,852 relations. UMLS gathered 1,887 alignments between ICD10 and MeSH. Only 1,606 of them were used for this study. Inter-alignment agreement using only validated MeSH to VCM alignment was 74.2% [70.5-78.0]_CI95%_, DSC_*crude*_ was 0.93 [0.91-0.94]_CI95%_, and DSC_*semantic*_ was 0.96 [0.95-0.96]_CI95%_. Discrepancy analysis revealed that even if two thirds of errors came from the reviewers, UMLS was nevertheless responsible for one third.

**Conclusions:**

This study has shown strong overall inter-alignment agreement between MeSH to VCM and ICD10 to VCM manual alignments. VCM icons have now been integrated into a guideline search engine (http://www.cismef.org) and a health terminologies portal (http://www.hetop.eu).

## Background

Finding pertinent medical information in a complex Electronic Health Record (EHR) [1,2] or inside guidelines [3] is a time-consuming task for physicians [4]. Visualization of Concepts in Medicine (VCM) is a compositional iconic language created by Lamy *et al.*[5] to ease this burden. VCM language has previously been used in a graphical interface for accessing drug knowledge, allowing physicians faster access to drug knowledge than with textual interface, and with fewer errors [6]. VCM can represent various signs, diseases, physiological states, risks, antecedents, drug and non-drug treatments, laboratory tests, and medical follow-up procedures by combining a small number of graphical primitives: colors, shapes and pictograms. For instance, the icon symbolizing “renal failure” is composed of a “kidney” pictogram, a downward arrow representing “diminished function”, and a red color standing for “current patient status”. VCM does not aim to achieve the same level of detail as natural language texts, but rather a broader level of detail. VCM icons can be used in medical applications for visually filtering information or for graphical summary. It has been implemented by Vidal®, the leader in drug databases in France, in its on-line guidelines^a^ and it is used by Sherbrooke Health Expertise Center for e-learning.

To allow this, the terminology used in the medical application has to be aligned to VCM language, i.e. each concept of the terminology has to be aligned to one or more VCM icon. For example, associating VCM icons with patient conditions coded in EHR with the tenth revision of the International Classification of Diseases (ICD10), requires iconic representation of each ICD10 code using VCM language. These alignments may also ease indexing and information retrieval, EHR visualization, as well as reading of Summary of Product Characteristics etc.

Alignment errors could lead to false display in the medical application and, possibly, to medical error. It is therefore important to limit these errors. The subjectivity of alignment [7] makes quality evaluation difficult and time-consuming. A potential method for performing such evaluation is inter-alignment agreement, as in indexing [8]. Several similarity metrics may be used to compare two alignments: icon comparison (are two icons identical?), elementary comparisons (is each compositional element of two icons identical?) and semantic comparison (do two icons share the same meaning?).

This study presents the alignment of two commonly used terminologies: ICD10 and Medical Subject Heading (MeSH), to VCM. The aim of this work was to evaluate alignment quality before integrating VCM in EHR. Based on a small proportion of MeSH to VCM alignment that had already been manually validated, three inter-alignment consistency measures were used: crude concordance and two measures based on Dice index, with or without semantics.

## Methods

### VCM iconic language (v2.07)

**Figure 1:**
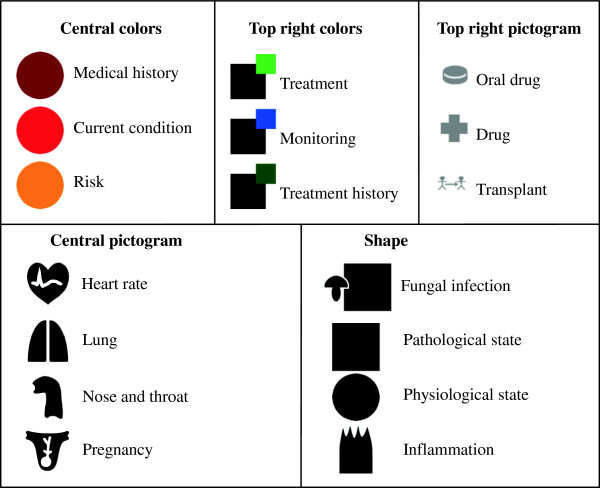
Examples of VCM primitives.

**Figure 2:**
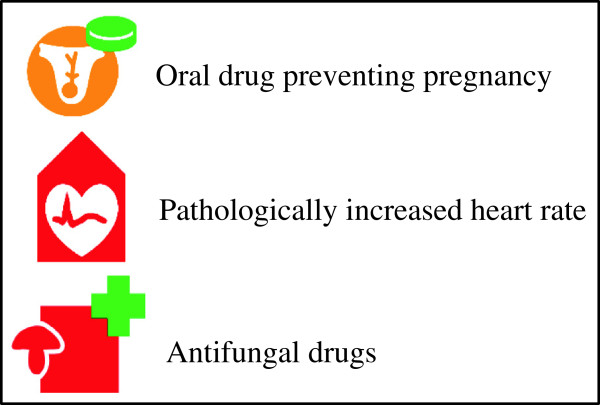
Some examples of VCM icons.

**Figure 3:**
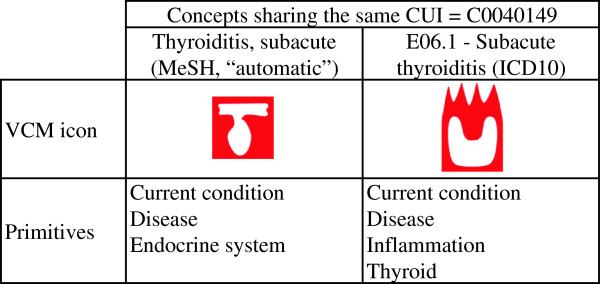
Primitive composition of VCM icons for two terms sharing the same CUI.

Each VCM icon is based on a combination of 7 components [5]. For each VCM icon, 5 out of 7 components determine the central color, the shape, the central pictogram, the top right color and the top right pictogram (see Figure 1). The two others are designed for a specific purpose and are not used in this study. Each component accepts a limited number of values called “primitives”, some of which allow multiple primitives. The use of combinatory grammar allows generation of billions of icons from these primitives (see Figure 2). Because of overlapping between some primitives, or nonsensical combinations, not all icons are allowed (such rules were formalized in [9]), but many are still valid. Note that all the components except central color and shape can be set to null. Primitives are organized hierarchically: the central pictograms of the examples in Figure 3 are linked by a “Broader Than – Narrower Than” relationship viz. the “Thyroid” central pictogram, which is a “child” of the “endocrine system” pictogram. As physicians do not have the time to learn complex iconic language, VCM has been designed to be learned in a few hours. Therefore, the hierarchy is very simple: 221 different primitives for a maximum of six levels. For a complete description of VCM language, interested readers may refer to: http://projet4-limbio.smbh.univ-paris13.fr/Joomla/.

### Reference terminologies

In this study, two reference terminologies were aligned to VCM iconic language: MeSH Thesaurus [10] of the US National Library of Medicine (NLM) in its 2011 version, mostly used for indexing and information retrieval of medical literature in MEDLINE, and the French version for Diagnosis Related Group of ICD10 [11], built for mortality statistics, but frequently used to code medical visits for budget allocation. These terminologies are widely used in the health domain.

The MeSH thesaurus has two different levels. The first one is the descriptor level, which is for users, and the focus of this work. It consists in a “small” (n ≈ 27,000) set of terms used for indexing and information retrieval. The second one is the concept level: each MeSH descriptor is the union of one or more MeSH concepts (n ≈ 50,000^b^). MeSH concept meaning may differ slightly from MeSH descriptors. It is a poly-hierarchic thesaurus, whereas ICD10 is a mono-hierarchic classification.

The V2010AB of the Unified Medical Language System Metathesaurus (UMLS) [12] was also used for this study. It is an NLM project that integrates several health terminologies and ontologies. Terms belonging to different terminologies but sharing the same meaning are gathered under the same Concept Unique Identifier (CUI). ICD10 and MeSH are both integrated into the UMLS and some concepts shared the same CUI.

All terminologies used here (including VCM), as well as their relationships, are accessible via the Health Terminologies/Ontology Portal (HeTOP; URL: http://www.hetop.eu) [13,14].

### Alignments between terminologies

#### MeSH descriptor to VCM alignment

Automatic approaches were first used to align MeSH to VCM. Natural language processing, stemming [15] and lemmatization techniques were tried but led to disappointing results. Only 1.6% of MeSH descriptors of interest were aligned. It was therefore necessary to perform this alignment manually. This task was performed by GK [16], a medical librarian. It was an iterative process leading to the addition of new icons and guidelines regarding VCM use.

**Figure 4:**
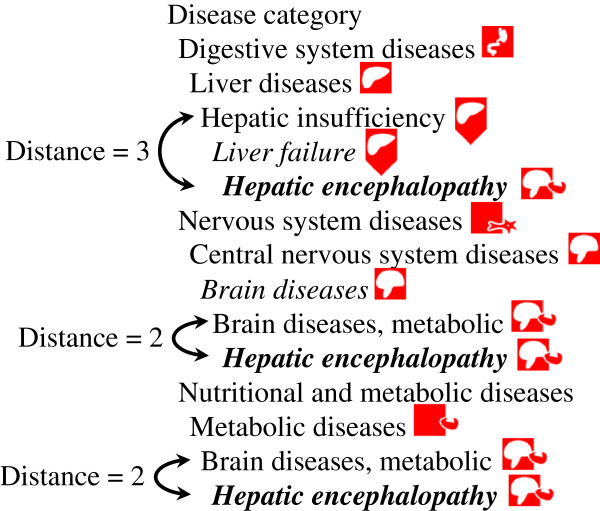
**Relationships between MeSH and VCM icons.** A short insight into MeSH hierarchy: Italic terms are automatically aligned with VCM whereas other terms are manually aligned. “Hepatic encephalopathy” (bold) inherits its icons from the closest parent (path length) manually aligned: “Brain diseases, metabolic”.

Some categories of the MeSH thesaurus, such as names of living organisms or geographical names, were not taken into account because they were outside of the scope of VCM. Every MeSH descriptor within a relevant category was examined and manually aligned to a VCM icon. During this process, if the expert considered that all sons of one term should share the same icons as the father, they inherited it. Problems arose when one son had many fathers: then an automated algorithm assigned it VCM icons from its closest parent, using a simple node counting scheme (see Figure 4). This resulted in two different types of relationships between VCM icons and MeSH descriptors: manual vs. automatic. Each manual alignment was reviewed by at least one of the VCM designers (JBL, CD and AV). The final alignment was obtained by consensus. This alignment allowed the use of VCM in a clinical guideline search engine [17].

#### ICD10 to VCM alignment

NG, a public health resident, performed ICD10 to VCM alignment. Each ICD10 code was manually aligned to VCM.

#### Alignment between MeSH and ICD10

To compare VCM icons aligned to MeSH and VCM icons aligned to ICD10, alignment between MeSH and ICD10 was necessary. The latter was provided by UMLS, and more specifically by selecting ICD10 codes and MeSH descriptors sharing the same CUI [18].

### Evaluation

Only manual MeSH to VCM alignments were already validated, and used to evaluate ICD10 to VCM alignments, which could in turn be used to validate automatic MeSH to VCM alignments. For each alignment between MeSH and ICD10, the following information was extracted: the MeSH descriptor, the relationship between the MeSH descriptor and the VCM icon, the VCM icon aligned to the MeSH descriptor, the ICD10 code, and the VCM icon aligned to the ICD10 code. Only alignments concerning one VCM icon for both ICD10 codes and MeSH descriptors were used, because of difficulties comparing more than two icons. Therefore, if one ICD10 code or one MeSH descriptor was aligned to more than one VCM icon, it was discarded from the study.

### Measuring inter-alignment agreement

Concordance was defined as the proportion of alignments in which the ICD10 code icon and the MeSH descriptor icon were identical. To refine this rough measure of inter-alignment agreement, the Dice Similarity Coefficient (DSC) [19] was used to compare icons based on their primitives. DSC is equivalent to Fleiss’ positive specific agreement [20], and as there are many primitives (n = 221), it is also equivalent to kappa coefficient [21,22].

Two DSC were calculated: a crude one (*DSC*_*crude*_) and a semantic one (*DSC*_*semantic*_). *DSC*_*crude*_ strictly compared VCM icon primitives, whereas *DSC*_*semantic*_ took meaning into account. *DSC*_*crude*_ was computed as follows:

(1) $$\mathrm{DS}C_{\mathrm{crude}}\left(I_{1},I_{2}\right)=\frac{2\times \left|\mathrm{Pr}\left(I_{1}\right)\cap \mathrm{Pr}\left(I_{2}\right)\right|}{\left|\mathrm{Pr}\left(I_{1}\right)\right|+\left|\mathrm{Pr}\left(I_{2}\right)\right|}$$

where *Pr(I*_*j*_*)* is the set of primitives for icon *I*_*j*_.

*DSC*_*semantic*_ was calculated combining the *DSC*_*crude*_ equation (1) with Lin semantic similarity [23]:

(2) $$\mathrm{sim}\left({\mathrm{Pr}}_{i},{\mathrm{Pr}}_{j}\right)=\frac{2\times \max_{\mathrm{Pr}\in S\left({\mathrm{Pr}}_{i},{\mathrm{Pr}}_{j}\right)}\left\{\log\left[p\left(\mathrm{Pr}\right)\right]\right\}}{\log\left[p\left({\mathrm{Pr}}_{i}\right)\right]+\log\left[p\left({\mathrm{Pr}}_{j}\right)\right]}$$

Where S(Pr_i_,Pr_j_) represents the set of ancestor primitives shared by both Pr_i_ and Pr_j_, “max” represents the maximum operator, and p(Pr) is the probability of finding Pr in a reference corpus (here, the probability of finding Pr as a primitive in the entire set of MeSH to VCM and ICD10 to VCM alignment). Lin similarity lies between 0 (when the only common ancestor is the root tree) and 1 (when Pr_i_ = Pr_j_).

To compute *DSC*_*semantic*_, the numerator of equation (1) is replaced by Lin semantic similarity: the presence of a primitive in the intersection between the two sets of primitives is replaced by the best semantic similarity between this primitive and the set of primitives for the other icon [24]. *DSC*_*semantic*_ formula is:

(3) $$\mathrm{DS}C_{\mathrm{semantic}}\left(I_{1},I_{2}\right)=\frac{\sum_{i}\max_{j}\left[\mathrm{sim}\left({\mathrm{Pr}}_{i},{\mathrm{Pr}}_{j}\right)\right]+\sum_{j}\max_{i}\left[\mathrm{sim}\left({\mathrm{Pr}}_{i},{\mathrm{Pr}}_{j}\right)\right]}{\left|{\mathrm{Pr}}_{I_{1}}\right|+\left|{\mathrm{Pr}}_{I_{2}}\right|}$$

Where sim(Pr_i_,Pr_j_) is computed using equation (2), and *i* and *j* are the number of primitives in I_1_ and I_2_, respectively.

The three metrics (*DSC*_*semantic*_, *DSC*_*crude*_ and concordance) ranged from 0 to 1, two identical icons having a DSC of 1. Figure 3 shows the primitives which composed the VCM icons corresponding to CUI C0040149 “Subacute thyroiditis”. Intersection and best similarities between these primitives are shown in Table 1.

**Table 1:** Computing DSC

	**Primitives**		**Similarity**	
	**Thyroiditis, subacute (MeSH)**	**E06.1 - Subacute thyroiditis (ICD10)**	**Crude**	**Semantic**
Best similarities for “MeSH primitives”	Current condition	Current condition	1	1
	Disease	Disease	1	1
	Endocrine system	Thyroid	0	0.85
Best similarities for “ICD10 primitives”	Current condition	Current condition	1	1
	Disease	Disease	1	1
	Disease	Inflammation	0	0.35
	Endocrine system	Thyroid	0	0.85
Total numerator			4	6.05
DSC			4/7	6.05/7

For these two different icons, *DSC*_*crude*_ = 4/7 and *DSC*_*semantic*_ = 6.05/7.

The three metrics were compared between icons according to the relationship between MeSH descriptors and VCM icons (automatic vs. manual), using Wilcoxon/Fisher tests.

### Discordance analysis

A random sample of 35 discordances, involving MeSH descriptors that were manually aligned to VCM, has been reviewed by experts (GK and NG) to assess the reasons for discordance.

## Results

### Alignments

Alignment from MeSH to VCM was performed manually for 1,830 MeSH descriptors and automatically (according to MeSH hierarchies) for 8,953 MeSH descriptors. It was not possible to measure the time spent performing this alignment since it was part of the evolution process of VCM. Alignment from ICD10 to VCM was totally manual. It took almost 70 hours to manually align the 19,852 ICD10 codes to VCM icons (see Table 2 for summary statistics concerning these alignments).

**Table 2:** Number of VCM icons by ICD10 code or MeSH descriptor, according to the relationship

		**VCM icons (N)**							**Total**
		**1**	**2**	**3**	**4**	**5**	**6**	**7**	
ICD10 code		12,966	6,005	838	38	5	0	0	19,852
MeSH descriptor	All	9,385	1,070	262	55	8	2	1	10,783
	Manual	1,794	36	0	0	0	0	0	1,830
	Automatic	7,591	1,034	262	55	8	2	1	8,953

How to read: MeSH descriptors with iconic representation = 10,783; Including 1,830 with manual representation, 1,794 of which are represented by 1 VCM icon, the other one is represented by 2 VCM icons.

There were 1,887 alignments between ICD10 and MeSH using UMLS concepts. For 1,606 of them, there was one icon for the ICD10 code and one icon for the MeSH descriptor (85.1%). This study focused on these 1,606 concepts, since comparing more than two icons would have been too complex. There were 528 manual alignments and 1,078 automatic alignments between MeSH descriptors and VCM icons.

### Inter-alignment agreement

Figure 3 shows an example of disagreement between two terms sharing the same CUI: “Thyroiditis, subacute” from MeSH and “Subacute thyroiditis” from ICD10.

Comparing MeSH Descriptor icons with ICD10 code icons showed that agreement differed according to alignment between VCM and MeSH. For all metrics, “manual” relationships were significantly better than “automatic” ones (see Table 3).

**Table 3:** Results from comparison of ICD10 code VCM icons and MeSH descriptor VCM icons

**MeSH to VCM relationship**	**Total (n = 1,606)**		**Manual (n = 528)**		**Automatic (n = 1,078)**		**p**
	**Mean/%**	**[CI**_**95%**_**]**	**Mean/%**	**[CI**_**95%**_**]**	**Mean/%**	**[CI**_**95%**_**]**	
Concordance	65%	[62.6-67.3]	74.2%	[70.5-78.0]	60.5%	[57.5-63.4]	* < 10^-4^
DSC_Crude_	0.90	[0.89-0.90]	0.93	[0.91-0.94]	0.88	[0.87-0.89]	^$^ < 10^-4^
DSC_Semantic_	0.93	[0.93-0.94]	0.96	[0.95-0.96]	0.92	[0.92-0.93]	^$^ < 10^-4^

*: Fisher test. ^$^: Wilcoxon test.

### Discordance analysis

**Figure 5:**
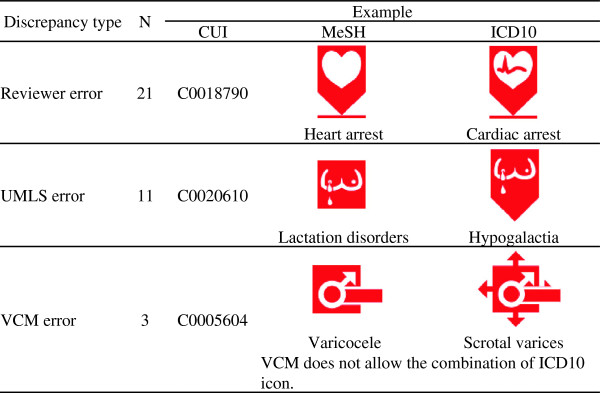
Analysis of discrepancies (n = 35).

Reviewing discordances between the MeSH expert and the ICD10 expert revealed that most errors came from the experts (60.0%; [44–76]_95% CI_), almost equally from the ICD10 expert (31.4%; [16–47]_95% CI_) and the MeSH expert (37.1%; [21–53]_95% CI_; for some alignments, both experts were wrong). These errors consisted, in general, in a lack of specificity: no icons were wrong, but one was more precise than the other. Nevertheless, as shown in Figure 5, the UMLS was also responsible for an important part of the discrepancies (31.4%; [16–47]_95% CI_). Lastly, few errors were caused by VCM itself: a lack of definition in primitives induced one error, and a lack of coherence in VCM’s rules of formalism led to two errors. See Additional file 1 for a complete description of discrepancies.

## Discussion

Inter-alignment agreement showed a concordance of 74.2% for fully manual alignments. The results are even better using Dice Similarity Coefficient: mean DSC_Crude_ = 0.93 and mean DSC_Semantic_ = 0.96. Both can be interpreted, like Cohen’s Kappa, as excellent [25] or almost perfect [26]. The results are less satisfying with automatic alignments: concordance dropped to 60.5%, and there was a decrease in both DSC to 0.88 and 0.92 respectively. Discordance analysis shows that discrepancies resulted mostly from experts (60%) or UMLS (31%).

Comparing automatic alignment to gold standard alignment (manually created by an expert) is frequent in the literature [27,28]. Conversely, few studies to date have compared two manually created alignments. Wieteck [29] compared inter-alignment agreement between two nursing terminologies: the European Nursing care Pathway, which is mono-axial, and the International Classification for Nursing Practice (ICNP), which is multi-axial. Agreement was measured for each of the eight ICNP axes and ranged from 73% to 100%. This led to an estimated overall inter-alignment agreement ranging from 53% to 70% for fully manual alignment. The results presented here are better than Wieteck’s [29] for manual alignment, especially for similarity metrics.

One explanation for these improved results could be the relatively low granularity of VCM iconic language with a maximum of six hierarchy levels, whereas the MeSH thesaurus has a maximum of 11 hierarchy levels. Nevertheless, the compositionality of VCM allows the creation of more icons than existing MeSH terms: according to VCM ontology, there are millions of coherent, consistent icons. This does not mean that each of these icons is meaningful. Today, more than 2,500 different icons have been created and linked to MeSH, ICD10, ATC or SNOMED.

Analysis of discrepancies revealed that alignment differences between VCM to ICD10 and VCM to MeSH may be the result of:

Firstly, VCM to MeSH alignment was performed by a medical librarian (GK), whereas VCM to ICD10 alignment was performed by a medical resident (NG). Consequently, alignment differences could be explained by different education and point of view regarding the disease. The purpose of the semantic similarity measure (DSC_*semantic*_) is to decrease the weight of such differences.

Secondly, sharing the same UMLS CUI is sometimes questionable based on the different contexts that led to the creation of the different terminologies (e.g. medical literature for MeSH, mortality statistics for ICD10) [30]. It is often the result of UMLS CUI linking an ICD10 code and a MeSH concept with narrower meaning than the MeSH descriptor used in this study. Nevertheless, those approximate links provide results of similar quality to more regular links, i.e. when MeSH concept and MeSH descriptor have exactly the same meaning (data not shown).

Lastly, differences in alignment could be explained by the different contexts of terminology in current use (e.g. billing for ICD10, indexing and information retrieval for MeSH).

This study has potential limitations. Firstly, it was based on a rather uncommon situation, with three different coexisting manual alignments: (1) MeSH to ICD10 alignment through UMLS (same CUI), (2) VCM to MeSH alignment and, (3) VCM to ICD10 alignment. VCM to MeSH alignment was performed first, then VCM to ICD10 thereafter. NG was not totally blind in performing the VCM to MeSH alignment. In case of doubt, he was able to use HeTOP [13,14], which had integrated VCM to MeSH alignment. Overall, the portal was used for a limited number of alignments. Such bias could therefore be considered as minimal. A second possible source of bias was the exclusion of ICD10 to MeSH alignment when more than one VCM icon was used for MeSH descriptor or for ICD10 code. Agreement in these cases might be lower than that observed here. However, from the 281 alignments concerned (i.e. MeSH descriptor or ICD10 code aligned to more than one VCM icon), only 42 involved an already validated MeSH to VCM alignment – i.e. manual MeSH to VCM alignment. Assuming those 42 were all erroneous, this would have led to a concordance of 68.8%, a DSC_crude_ of 0.86 and a DSC_semantic_ of 0.89. It is still an excellent inter-alignment agreement, especially compared to the literature. Lastly, our results concerned only about 20% of MeSH diseases and 10% of ICD10. Those terms were not chosen randomly but rather based on whether they were mappable to a UMLS CUI that was also mapped to the other terminology. Also, the remaining terms may have some systematic characteristics: being more specific, with nuances that make them incomplete matches etc. This implies that for those terms alignment to VCM might require more work, more detailed icons (with more primitives) and therefore be more prone to coder errors, show lower levels of concordance, similarity and, finally, validity. Such differences between UMLS linked and non-UMLS linked MeSH descriptors and ICD10 codes are difficult to quantify.

For research and development purposes, both alignments will be maintained in HeTOP, allowing VCM to MeSH available in 16 languages (e.g. Japanese and Swedish) and VCM to ICD10 in 11 languages (e.g. Arabic and Italian). However, industrial partners in the L3IM consortium [31] (one small French company and one French subsidiary of a north-American company) have different perspectives: the same medical concept should have the same VCM icon for the end-user, no matter which terminology or classification it was aligned from. Such recommendations require a considerable amount of expert validation and, probably, some changes in VCM hierarchy.

The high inter-alignment agreement involving already validated MeSH to VCM alignments demonstrates the validity of ICD10 to VCM alignment, allowing its use in ICD10 based EHR to summarize patient conditions, with minor modification from editors. Two companies have already shown enough interest in VCM to introduce it in their products (Silk [32] and McKesson). VCM can therefore be considered as a sort of interface terminology, which was defined by Rosenbloom *et al*. [33] as a terminology that “facilitates display of computer-stored patient information to clinician-users as simple human-readable text”.

The literature suggests that enhanced consistency between MeSH to VCM and ICD10 to VCM alignment could increase alignment validity [8]. Therefore, finding an approach for MeSH to VCM automatic alignment leading to consistency similar to that found in “manual” relationship would probably facilitate validation of industrial recommendations. L3IM intends working on such an approach using the ontological version of VCM iconic language [9].

## Conclusion

This study has shown excellent overall inter-alignment semantic agreement between MeSH to VCM and ICD10 to VCM manual alignments. ICD10 to VCM alignment seems of sufficient quality to be used in medical applications.

## Endnotes

^a^See http://www.vidal.fr/recommandations/3398/diverticulose_colique/la_maladie/, for example.

^b^Excluding MeSH supplementary concepts, which are not used for this study.

## Abbreviations

ATC: Anatomical therapeutic chemical classification system; CUI: Concept Unique Identifier; DSC: Dice Similarity Coefficient; EHR: Electronic Health Records; HeTOP: Health terminology/ontology portal; ICD10: International Classification of Diseases – tenth revision; ICNP: International Classification for Nursing Practice; L3IM: Iconic language and interactive user interfaces in medicine; MeSH: Medical Subject Headings; NLM: National Library of Medicine; SNOMED: Systematized NOmenclature of MEDicine; UMLS: Unified Medical Language System; VCM: Visualization of Concepts in Medicine.

## Competing interests

The authors declare that they have no competing interests. VCM language is protected by an international patent taken out by Paris 13 University.

## Authors’ contributions

NG made VCM to ICD10 alignment, performed comparisons and statistical analysis and drafted the manuscript with LFS. GK made MeSH to VCM alignment and helped to write the manuscript. SJD conceived the study and helped to write the manuscript. AV, JBL and CD created VCM and validated VCM to MeSH alignment. TM extracted UMLS relationship. JG implemented VCM alignments into HeTOP. All the authors approved the manuscript.

## Pre-publication history

The pre-publication history for this paper can be accessed here:

http://www.biomedcentral.com/1472-6947/14/17/prepub

## Supplementary Material

Additional file 1 **Summarizes all the ICD10/MeSH couples with discordant icons that were analyzed.** MeSH descriptor and ICD10 code on the same line share the same UMLS CUI but do not have the same VCM icon. Click here for file
