# Supplementary material for: Evaluating alignment quality between iconic language and reference terminologies using similarity metrics
Source: BMC Med Inform Decis Mak. 2014 Mar 11;14:17. doi: 10.1186/1472-6947-14-17 (PMC4007774; doi:10.1186/1472-6947-14-17)
Supplement: Additional file 1 — Summarizes all the ICD10/MeSH couples with discordant icons that were analyzed. MeSH descriptor and ICD10 code on the same line share the same UMLS CUI but do not have the same VCM icon. [file 1472-6947-14-17-S1.pdf]

Discrepancies

The following table summarizes all the ICD10/MeSH couples with discordant icons that were analyzed. MeSH descriptor and ICD10 code on the same line share the same UMLS CUI but do not have the same VCM icon. The discrepancy type was assessed by both aligners (GK and NG), on consensus.
The corresponding icons can be seen in the Health Terminology/Ontology Portal (www.hetop.eu)

MeSH descriptor	ICD10 code	Discrepancy type
Intestinal diseases, parasitic	Unspecified intestinal parasitism	Reviewer error
Radiodermatitis	Radiodermatitis	Reviewer error
arthritis, infectious	Pyogenic arthritis	UMLS error
Intestinal diseases, parasitic	Intestinal parasitism, unspecified	Reviewer error
Spinal cord neoplasms	Spinal cord	UMLS error
Radiodermatitis	Radiodermatitis, unspecified	Reviewer error
arthritis, infectious	Pyogenic arthritis, unspecified	UMLS error
Myositis	Infective myositis	UMLS error
Urinary tract infections	Urinary tract infection, site not specified	Reviewer error
Abortion, threatened	Threatened abortion	Reviewer error
Lactation disorders	Hypogalactia	UMLS error
Birth injuries	Birth injury, unspecified	VCM error
Skin abnormalities	Congenital malformation of integument, unspecified	Reviewer error
Alcohol drinking	Alcohol use	UMLS error
Heart arrest	Cardiac arrest	Reviewer error
Heart arrest	Cardiac arrest, unspecified	Reviewer error
Urologic diseases	Disorder of urinary system, unspecified	Reviewer error
Optic nerve neoplasms	Optic nerve	Reviewer error
Rheumatic diseases	Rheumatism, unspecified	Reviewer error
Endometriosis	Endometriosis	Reviewer error
Cellulitis	Cellulitis, unspecified	Reviewer error
Facial nerve diseases	Geniculate ganglionitis	UMLS error
Colitis, ulcerative	Ulcerative colitis	UMLS error
Sex offenses	Sexual abuse	UMLS error
Varicocele	Scrotal varices	VCM error
Pemphigoid, bullous	Bullous pemphigoid	Reviewer error
Infectious mononucleosis	Infectious mononucleosis	VCM error
Erythema	Erythematous condition, unspecified	Reviewer error
Ocular motility disorders	Internuclear ophthalmoplegia	UMLS error
Craniocerebral trauma	Superficial injury of head, part unspecified	UMLS error
Reflex, abnormal	Abnormal reflex	Reviewer error
Craniocerebral trauma	Unspecified injury of head	Reviewer error
Staphylococcal food poisoning	Foodborne staphylococcal intoxication	Reviewer error
Dermatitis, atopic	Atopic dermatitis, unspecified	Reviewer error
Hemothorax	Haemothorax	Reviewer error
